# Effects of *L*-lysine·H_2_SO_4_ product on the intestinal morphology and liver pathology using broiler model

**DOI:** 10.1186/s40104-019-0318-9

**Published:** 2019-02-07

**Authors:** Hongmin Jia, Ting He, Haitao Yu, Xiangfang Zeng, Shihai Zhang, Wenfeng Ma, Jie Zhang, Shiyan Qiao, Xi Ma

**Affiliations:** 1 State Key Laboratory of Animal Nutrition, College of Animal Science and Technology, China Agricultural University, Beijing, 100193 China; 2 Guangdong Provincial Key Laboratory of Animal Nutrition Control, College of Animal Science, South China Agricultural University, Guangzhou, 510642 China; 3 College of Animal Science and Technology, Henan University of Science and Technology, Luoyang, 471003 Henan China; 4 College of Animal Science and Technology, Qingdao Agricultural University, Qingdao, 266109 China

**Keywords:** Broilers, Intestinal morphology, Liver pathology, *L*-lysine·H_2_SO_4_

## Abstract

**Background:**

Lysine is used widely in livestock production due to the shortage of feed protein resources. *L*-lysine·H_2_SO_4_ contains *L*-lysine sulphate as well as fermentation co-products which contain other amino acids and phosphorus. However, there are few articles about *L*-lysine·H_2_SO_4_ product regarding intestinal morphology and liver pathology of broiler chickens. In this article, we focus on the absorption and metabolism of *L*-lysine·H_2_SO_4_ revealed in the variation of intestinal morphology and liver pathology to determine the tolerance of chicks for *L*-lysine·H_2_SO_4_.

**Methods:**

To evaluate the tolerance of broilers for *L*-lysine·H_2_SO_4_, 240 one day old broilers were allocated randomly to one of five dietary treatments which included corn-soybean diets containing 0, 1%, 4%, 7% or 10% *L*-lysine·H_2_SO_4_ (*L*-lysine content = 55%).

**Results:**

Supplementation of 1% *L*-lysine·H_2_SO_4_ in the diet had no negative effects. However, 4%, 7% or 10% *L*-lysine·H_2_SO_4_ supplementation produced negative responses on broiler performance, carcass characteristics, blood biochemistry, and particularly on intestinal morphology and liver pathology compared with broilers fed the control diet.

**Conclusion:**

Our results show that supplementation with 1% *L*-lysine·H_2_SO_4_ had no negative effects on performance, carcass characteristics, blood biochemistry, intestinal morphology and liver pathology in broilers, but supplementation with 4%, 7% or 10% *L*-lysine·H_2_SO_4_ produced a negative response, particularly with respect to intestinal morphology and liver pathology.

## Background

Lysine is a limiting amino acid that is commonly supplemented in the form of *L*-lysine·HCl in diets fed to pigs [1, 2], rainbow trout [3], and broiler chickens [4]. In recent years, an alternative source of lysine in the form of *L*-lysine·H_2_SO_4_ (55% lysine) has been developed with its use being highly attractive for both ecological and economic reasons. This occurs because the production of L-lysine·HCl involves a costly, multiple-stage process requiring the handling of hazardous materials such as ammonia solution (elution of the Ion exchange column) and hydrochloric acid (neutralization of the *L*-lysine base) which generates residual solutions that are harmful to the environment [5, 6].

*L*-lysine·H_2_SO_4_ is a new source of lysine containing *L*-lysine·sulphate and fermentation co-products which contain other amino acids and phosphorus. This new source of lysine has been shown to be equally efficacious compared with *L*-lysine·HCl in diets fed to broilers [6, 7]. Furthermore, the mortality of broiler chickens fed *L*-lysine·H_2_SO_4_ was lower from d 1 to d 42 compared with birds fed *L*-lysine·HCl, which suggests that *L*-lysine·H_2_SO_4_ can improve the bird’s immune status.

Lysine is an indispensable amino acid for non-ruminant animals and has been widely used as a supplement in livestock diets [8, 9]. However, excessive concentrations of dietary lysine can produce negative effects in chickens, such as decreased weight gain, and increased incidence of severe leg problems [10–12]. Furthermore, there are no reports about the effects of high levels of *L*-lysine·H_2_SO_4_ on performance, carcass characteristics, blood biochemistry, intestinal morphology or liver pathology of broiler chickens. Therefore, we designed this experiment to determine the tolerance of broiler chickens to *L*-lysine·H_2_SO_4_ and observe the effect of this product on intestinal morphology and liver pathology when used as a source of lysine in diets.

## Methods

### Experimental material

*L*-lysine·H_2_SO_4_ (*L*-lysine content = 55%) was manufactured by the Changchun Dacheng Industrial Group (Changchun, China).

### Birds and experimental design

All management and experimental procedures followed the animal care protocol approved by the Animal Care and Use Committee of China Agricultural University (Beijing, China). This study was conducted on Arbor Acres broiler chickens (Beijing, China) for a 35-day period with a completely randomized design. One day old (*n* = 240) broiler chickens were housed in a standard broiler house with separate feed and drinking facilities. These broilers were weighed individually, wing banded, and allocated randomly to 1 of 5 treatments with 8 replicates per treatment and 6 birds per replicate.

The corn-soybean meal diets were formulated as shown in Table 1. The experimental diets were comprised of a control diet with no supplemental lysine and 4 diets supplemented with 1%, 4%, 7% or 10% *L*-lysine·H_2_SO_4_ in two periods (first period: d 1 to 21, second period: d 22 to 35). According to the “European Food Safety Authority” [13], “Guidelines for the Evaluation of Tolerance of Feed and Feed Additives on Livestock and Poultry Target Animals” (2011) and “Feeding Standard for Poultry” (2004), the multidose group is generally 10 times as the effective dose for evaluating new feed products. As prescribed, the general addition of lysine in feed was 1%. Thus, 1% was selected as the initial concentration, and increased by 3% points in turn, reaching the highest concentration of 10%. Meanwhile, 10% as the highest dose for lysine was also consistent with the standard. The control diet provided 3.04 Mcal/kg according to the nutrient requirements for chickens.

**Table 1:** Ingredient composition and nutrient levels of the experimental diets (% as fed)

Items	Dietary *L*-lysine·H_2_SO_4_ levels (%) fed from d 1 to 21					Dietary *L*-lysine·H_2_SO_4_ levels (%) fed from d 22 to 35				
	0	1	4	7	10	0	1	4	7	10
Ingredient, %										
Corn	59.12	59.75	62.41	65.21	68.85	60.67	61.27	63.67	67.77	71.58
Soybean meal	31.00	29.40	24.00	19.95	14.65	31.75	30.10	24.45	18.34	12.43
Fish meal	3.25	3.15	2.95	1.55	1.00	0.00	0.00	0.00	0.00	0.00
Soybean meal	3.00	2.97	2.80	2.00	1.00	4.00	4.00	3.95	2.70	1.70
Limestone	1.40	1.46	1.35	1.30	1.25	1.10	1.15	1.20	1.25	1.20
Dicalcium phosphate	1.10	1.12	1.20	1.50	1.60	1.40	1.40	1.52	1.55	1.55
Salt	0.30	0.30	0.30	0.30	0.30	0.30	0.30	0.30	0.30	0.30
Mineral and vitamin premix^1^	0.50	0.50	0.50	0.50	0.50	0.50	0.50	0.50	0.50	0.50
*L*-lysine·H_2_SO_4_	0.00	1.00	4.00	7.00	10.00	0.00	1.00	4.00	7.00	10.00
*DL*-methionine	0.15	0.16	0.20	0.25	0.28	0.10	0.10	0.15	0.20	0.21
*L*-tryptophan	0.00	0.00	0.00	0.04	0.07	0.00	0.00	0.00	0.02	0.06
*L*-threonine	0.00	0.01	0.11	0.22	0.32	0.00	0.00	0.08	0.19	0.29
Choline chloride	0.13	0.13	0.13	0.13	0.13	0.13	0.13	0.13	0.13	0.13
Antioxidant	0.05	0.05	0.05	0.05	0.05	0.05	0.05	0.05	0.05	0.05
Calculated nutritional value										
ME, Mcal/kg	3.04	3.05	3.10	3.10	3.10	3.10	3.11	3.16	3.15	3.16
Analyzed nutritional value, %^2^										
Dry matter	88.24	88.33	88.62	88.85	88.82	89.10	89.11	89.28	89.47	90.03
Crude protein	21.70	22.82	22.59	23.13	22.71	20.02	21.20	21.58	20.40	20.31
Total calcium	1.05	1.14	0.73	0.71	1.01	0.96	1.20	0.97	0.82	1.10
Total phosphorus	0.49	0.55	0.49	0.61	0.57	0.57	0.55	0.54	0.55	0.58
Lysine	1.30	1.89	3.18	4.19	5.78	1.25	1.69	3.57	4.61	6.47
Tryptophan	0.23	0.23	0.20	0.21	0.19	0.23	0.22	0.19	0.18	0.20
Methionine	0.49	0.49	0.49	0.48	0.43	0.36	0.38	0.42	0.42	0.39
Cystine	0.30	0.29	0.28	0.25	0.21	0.28	0.29	0.28	0.25	0.21
Threonine	0.81	0.80	0.83	0.83	0.80	0.78	0.75	0.73	0.75	0.77

^1^The mineral and vitamin premix provided the following per kg of feed: zinc, 75 mg; iron, 200 mg; copper, 15 mg; iodine, 0.88 mg; selenium, 0.35 mg; manganese, 100 mg; vitamin A, 9000 IU; vitamin D_3_, 3750 IU; vitamin E, 25 IU; vitamin K_3_, 2 mg; thiamine, 1.5 mg; vitamin B_12_, 120 μg; riboflavin, 6 mg; nicotinic acid, 40 mg; pantothenic acid, 10 mg; pyridoxine, 3 mg; biotin, 0.1 mg

^2^Values are the means of a chemical analysis conducted in duplicate, *n* = 6/diet

### Experimental management

The broiler chickens were kept in zinc-plated cages (90 cm × 70 cm × 50 cm) with plastic slatted flooring. The chicks were housed in cages with 24 h constant-lighting and had free access to feed and water. All broilers were vaccinated with live, combined Newcastle disease and infectious bronchitis vaccine on d 7 and d 21 and inactivated infectious bursal disease vaccine on d 14 and d 28.

### Dietary analysis

Diets were analyzed for dry matter, crude protein, calcium, total phosphorus and amino acids according to AOAC [14] procedures. Lysine and threonine were determined by Ion-Exchange Chromatography using an AA Analyzer (Hitachi L-8800, Tokyo, Japan) after acid hydrolysis with 6 mol/L HCl (refluxed for 24 h at 110 °C). Methionine and cystine were determined after oxidation with performic acid and subsequent hydrolysis with 6 mol/L HCl at 120 °C for 16 h and separated by Reversed-Phase HPLC (Agilent 1200, Santa Clara, CA). Tryptophan was measured after LiOH hydrolysis for 22 h at 110 °C using High-Performance Liquid Chromatography (Agilent 1200 Series, Santa Clara, CA).

### Growth performance

Broilers were weighed on d 1, 21, and 35. Feed consumption on a pen was measured every week. Average daily feed intake (ADFI), average daily gain (ADG), and feed conversion ratio (FCR, ADFI: ADG) were calculated from d 1 to 21, d 22 to 35 and d 1 to 35, respectively.

### Carcass characteristics

On the d 35 of the trial, one broiler chicken was selected randomly from each cage after an overnight fast. The broilers were slaughtered by exsanguination and eviscerated manually. Carcass weight, semi-eviscerated weight, eviscerated weight, liver weight, abdominal fat weight and pH_45 min_ of the breast and thigh muscle were measured. Dressing percentage, semi-eviscerated percentage, eviscerated percentage, abdominal fat percentage and drip loss were calculated according to the Performance Terms and Measurement for Poultry (NY/T 823–2004, China). Drip loss was calculated by hanging a section (breast and thigh muscle sample) in an inflated and closed plastic bag for 24 h at 4 °C.

### Intestinal morphology and liver pathology

Samples of the duodenum, jejunum, ileum, and liver were dissected from the slaughtered broilers and immediately fixed in 4% (*v*/*v*) paraformaldehyde solution for histological examination. The fixed tissues were processed routinely, embedded in paraffin and cut into 5 μm serial sections, and sections from each tissue sample were selected and stained with hematoxylin–eosin for identification [15]. Five well-oriented villi (determined as the distance between the crypt openings and the end of the villi) and their associated crypt (measured from the crypt villous junction to the base of the crypt) per section were selected and measured under a light microscope (CK-40, Olympus, Tokyo, Japan) at 40 × magnification and analyzed with an Image Analyzer (Lucia Software. Lucia, Za Drahou, Czechoslovakia). The sections of liver were prepared for microscopic examination and representative samples were photographed.

### Blood biochemistry

Blood was collected in uncoated serum tubes and EDTA-coated tubes at 35 days of age from 8 birds in each treatment by puncturing the brachial vein. Serum was collected and stored at − 20 °C. Serum amino acid concentrations were determined with an Amino Acid Analyzer (S-433D AA Analyzer, Sykam, Germany). Individual serum samples were analyzed for albumin, total protein, globulin, total bilirubin, creatinine and uric acid. The activities of alanine amino transferase, aspartate amino transferase and lactic dehydrogenase were analyzed at the Beijing Sino-UK Institute of Biological Technology Company (Beijing, China). Hematology parameters such as white blood cell count, red blood cell count, hemoglobin, hematocrit, corpuscular volume, corpuscular hemoglobin concentration, red cell distribution width, platelet distribution width, platelet volume and proportion of large platelets were measured by corresponding commercial kits (BioSino Bio-Technology and Science Company, Beijing, China) using an Automatic Biochemical Analyzer (Hitachi 7160, Hitachi High-Technologies Corporation, Tokyo, Japan).

### Statistical analysis

Data were analyzed by the GLM procedure of SAS (Statistical Analysis System, Version 9.2) using a randomized design. Means are expressed as the least squares means with pen as the experimental unit. An alpha level of *P* < 0.05 was the criterion for statistical significance. Polynomial contrasts were performed to determine linear and quadratic relationships of lysine levels.

## Results

### Growth performance

Increasing dietary *L*-lysine·H_2_SO_4_ linearly and quadratically decreased both ADG and ADFI and increased FCR from d 1 to 21, d 22 to 35, and d 1 to 35 (*P* < 0.05) (Table 2). No significant difference in performance was observed between broiler chicks fed the control and 1% *L*-lysine·H_2_SO_4_ diets during any phase of the experiment (Table 2). When the *L*-lysine·H_2_SO_4_ level in the diet increased to 4%, 7% or 10%, the performance of broilers was negatively affected compared with broiler chicks fed the control and 1% *L*-lysine·H_2_SO_4_ diets during all phases of the experiment (*P* < 0.05) (Table 2).

**Table 2:** Effect of dietary *L*-lysine·H_2_SO_4_ levels on performance of broiler chickens^1^

Items	Dietary *L*-lysine·H_2_SO_4_ levels, %					SEM	*P-*value		
	0	1	4	7	10		ANOVA	Linear	Quadratic
D 1 to 21									
Average daily feed intake, g	43.0^a^	42.7^a^	32.9^b^	13.5^c^	6.5^d^	0.66	< 0.01	< 0.01	< 0.01
Average daily gain, g	32.7^a^	32.7^a^	22.6^b^	6.0^c^	1.5^d^	0.77	< 0.01	< 0.01	< 0.01
Feed conversion ratio	1.3^d^	1.3^d^	1.5^c^	2.3^b^	4.4^a^	0.06	< 0.01	< 0.01	< 0.01
D 22 to 35									
Average daily feed intake, g	111.3^a^	109.2^a^	56.5^b^	24.7^c^	10.8^d^	2.00	< 0.01	< 0.01	< 0.01
Average daily gain, g	67.0^a^	65.6^a^	19.5^b^	6.3^c^	1.9^d^	1.52	< 0.01	< 0.01	< 0.01
Feed conversion ratio	1.7^d^	1.7^d^	3.0^c^	4.1^b^	6.0^a^	0.26	<0.01	<0.01	<0.01
D 1 to 35									
Average daily feed intake, g	70.3^a^	69.3^a^	42.4^b^	18.0^c^	8.2^d^	1.00	< 0.01	< 0.01	< 0.01
Average daily gain, g	46.4^a^	45.9^a^	21.4^b^	6.1^c^	1.6^d^	0.83	<0.01	< 0.01	< 0.01
Feed conversion ratio	1.5^d^	1.5^d^	2.1^c^	3.0^b^	5.1^a^	0.12	< 0.01	<0.01	< 0.01

^1^Means in the same row with different superscripts significantly differ from one another (*P* < 0.05). *SEM* standard error of the mean; *n* = 8/diet

### Carcass characteristics

No significant difference in carcass characteristics was observed between broilers fed the control and 1% *L*-lysine·H_2_SO_4_ diets (Table 3). Increased dietary *L*-lysine·H_2_SO_4_ linearly and quadratically decreased eviscerated percentage and abdominal fat percentage of broilers (*P* < 0.05), and increased liver index and drip loss (breast and thigh muscle) of broiler chickens (*P* < 0.05). There were no notably changes in pH value among different levels of *L*-lysine·H_2_SO_4_ treatments.

**Table 3:** Effect of dietary *L*-lysine·H_2_SO_4_ levels on carcass characteristics of broiler chickens^1^

Items	Dietary *L*-lysine·H_2_SO_4_ levels, %				SEM	*P*-value		
	0	1	4	7		ANOVA	Linear	Quadratic
Dressing percentage, %	91.5	90.2	90.9	91.4	0.83	0.66	0.97	0.54
Semi-eviscerated percentage, %	86.0	84.2	83.4	81.9	1.11	0.12	0.01	0.53
Eviscerated percentage, %	73.4^a^	71.9^a^	67.6^b^	62.7^c^	1.30	< 0.01	< 0.01	< 0.01
Abdominal fat percentage, %	1.1^ab^	1.2^a^	0.8^b^	0.08^c^	1.13	< 0.01	< 0.01	< 0.01
Liver index, %	2.1^b^	2.1^b^	2.8^b^	4.3^a^	0.19	< 0.01	< 0.01	< 0.01
Breast muscle								
pH _45 min_	6.2	6.2	6.5	6.4	0.09	0.11	0.05	0.11
Drip loss, %	2.1^b^	2.2^b^	3.3^b^	6.7^a^	0.43	< 0.01	< 0.01	< 0.01
Thigh muscle								
pH _45 min_	6.5	6.5	6.6	6.7	0.06	0.05	0.01	0.02
Drip loss, %	3.3^b^	4.4^b^	4.2^b^	12.7^a^	0.91	< 0.01	< 0.01	< 0.01

^1^Broiler chickens fed with 10% *L*-lysine·H_2_SO_4_ supplemented diet were too small to collect carcass characteristics in this experiment. Means in the same row with different superscripts significantly differ from one another (*P* <  0.05). *SEM* standard error of the mean; n = 6/diet

### Intestinal morphology

**Fig. 1:**
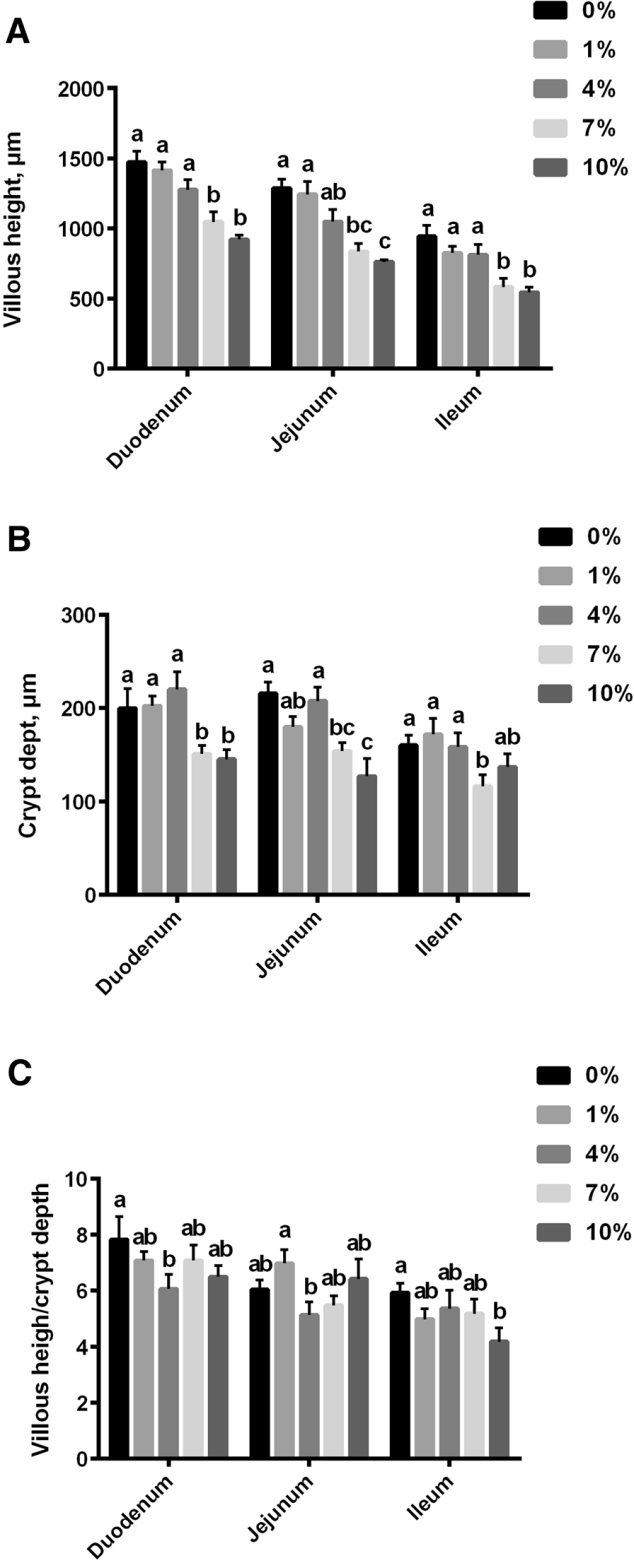
Effect of dietary *L*-lysine·H_2_SO_4_ levels (%) on intestinal morphology of broiler chickens. **a**, villous height (μm) of broiler chickens fed different diets; **b**, crypt depth (μm) of broiler chickens fed different diets; **c**, ratio of villous height to crypt depth of broiler chickens fed different diets. Data were means ± SEM (*n* = 6). Different letters indicate a difference (*P* <  0.05)

Intestinal morphology was influenced by high levels of dietary *L*-lysine·H_2_SO_4_ (Fig. 1), but no significant differences were observed between broilers fed control and 1% *L*-lysine·H_2_SO_4_ diets. Villous height and crypt depth in broilers fed the 7% and 10% *L*-lysine·H_2_SO_4_ diets were lower than those of the broilers fed 0, 1% and 4% *L*-lysine·H_2_SO_4_ diets (*P* < 0.05). Villous height, crypt depth and villous height to crypt depth ratio in the duodenum, jejunum, and ileum were not affected when comparing to broilers fed control with those fed 1% *L*-lysine·H_2_SO_4_ diets (*P* > 0.05).

### Liver pathology

**Fig. 2:**
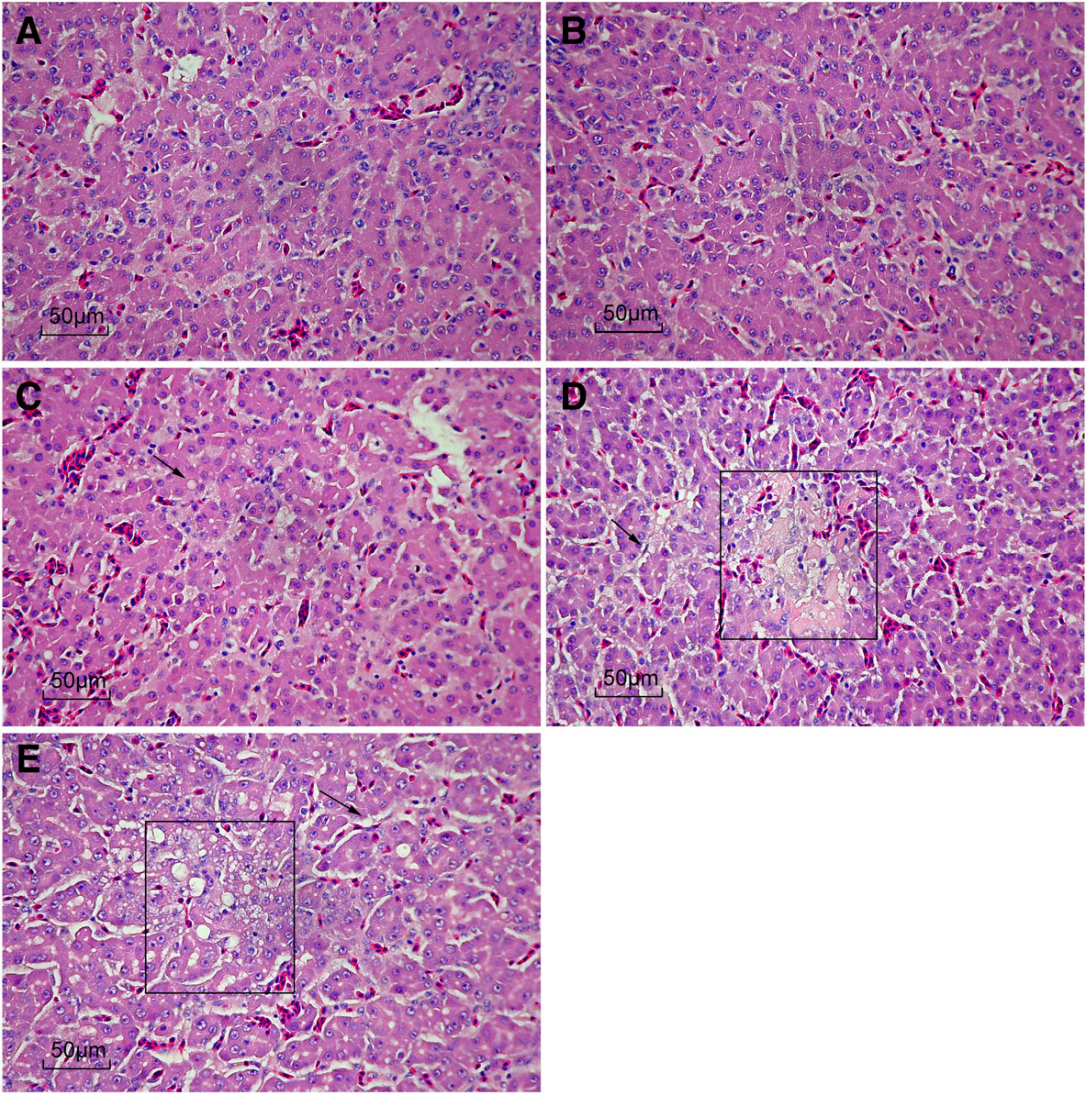
Effect of dietary *L*-lysine·H_2_SO_4_ levels on liver morphology of broiler chickens. **a**, Broilers fed on basal diets; **b**, Broilers supplemented with 1% *L*-lysine·H_2_SO_4_; **c**, Broilers supplemented with 4% *L*-lysine·H_2_SO_4_; **d**, Broilers supplemented with 7% *L*-lysine·H_2_SO_4_; **e**, Broilers supplemented with 10% *L*-lysine·H_2_SO_4_

No differences pathology of livers were observed between broilers fed the control and 1% *L*-lysine·H_2_SO_4_ diets (Fig. 2). Hepatocytes were arranged compactly and orderly. There were a few lipid droplets in hepatocytes of broilers fed the 4% *L*-lysine·H_2_SO_4_ diets. When broilers were fed 7% *L*-lysine·H_2_SO_4_, obvious pathological changes were present in those livers. Liver cells were dissociated from each other in hepatic cords, cytoplasmic fat vacuoles. Pushed hepatocyte nuclei to the cell periphery in some places and reduction in size of a few hepatocytes was also seen. There was diffuse infiltration of fat vacuoles indicating vacuolar degeneration in livers of broilers fed the 10% *L*-lysine·H_2_SO_4_ diet. Furthermore, liver cells were dissociated from each other and cytoplasmic fat vacuoles pushed hepatocyte nuclei to the cell periphery in many cells. Tissue edema was obvious in livers.

### Serum amino acid concentrations

No significant differences were observed among broilers fed control, 1%, 4%, 7% and 10% *L*-lysine·H_2_SO_4_ diets for serum concentrations of arginine, histidine, methionine, cysteine, serine and tyrosine (Table 4). Serum lysine and threonine concentrations of broilers were linearly and quadratically increased with increasing dietary *L*-lysine·H_2_SO_4_ level (*P* < 0.05). Serum glycine concentration of broilers fed 1% *L*-lysine·H_2_SO_4_ diet was higher than that of broilers fed control diets (*P* < 0.05), while serum proline concentration of the broilers fed 1% *L*-lysine·H_2_SO_4_ diet was higher than that of broilers fed the 4% *L*-lysine·H_2_SO_4_ diet (*P* < 0.05). Serum isoleucine, leucine and valine concentrations of the broilers fed the 7% *L*-lysine·H_2_SO_4_ diet was higher than that of broilers fed the control diet (*P* < 0.05).

**Table 4:** Effect of dietary *L*-lysine·H_2_SO_4_ levels on serum amino acid concentrations of broilers ^1^

Items	Dietary *L*-lysine·H_2_SO_4_ levels, %					SEM	*P*-value		
	0	1	4	7	10		ANOVA	Linear	Quadratic
Essential AA, nmol/mL									
Lysine	402.0^c^	576.5 ^c^	1031.4^b^	1395.3^a^	1521.8^a^	105.18	< 0.01	< 0.01	< 0.01
Arginine	374.9	310.3	281.7	319.4	284.4	43.55	0.58	0.21	0.37
Histidine	114.9	145.6	137.3	144.7	157.7	14.91	0.36	0.07	0.20
Isoleucine	112. 9^b^	170.9^ab^	189.0^ab^	231.9^a^	156.4^ab^	21.39	< 0.01	0.10	< 0.01
Leucine	175.8^b^	258.0^ab^	229.0^ab^	283.0^a^	228.3^ab^	24.59	0.04	0.12	0.04
Methionine	68.9	80.8	79.4	81.5	76.7	6.93	0.69	0.44	0.37
Phenylalanine	121.5^b^	166.7^a^	178.8^a^	176.5^a^	138.7^ab^	11.96	< 0.01	0.30	< 0.01
Threonine	623.5^b^	667.7^b^	776.7^b^	1070.5^b^	1453.0^a^	117.63	< 0.01	< 0.01	< 0.01
Valine	169.4^c^	260.9^b^	312.9^b^	408.6^a^	310.1^b^	27.71	< 0.01	< 0.01	< 0.01
Non-essential AA, nmol/mL									
Alanine	1043.5^b^	1797.6^ab^	806.1^b^	2167.4^a^	848.9^b^	258.68	< 0.01	0.92	0.23
Asparagine	79.9^b^	182.1^ab^	118.8^ab^	221.3^a^	143.4^ab^	30.28	0.02	0.11	0.07
Glycine	764.9^b^	1058.2^a^	918.5^ab^	911.0^ab^	845.5^ab^	66.07	0.05	0.95	0.09
Cysteine	304.9	403.0	296.5	415.1	238.6	53.94	0.12	0.57	0.22
Proline	1926.8^ab^	2622.0^a^	1464.6^b^	1881.1^ab^	2415.6^a^	204.41	< 0.01	0.80	0.50
Serine	647.1	764.4	601.3	656.5	751.3	58.40	0.27	0.63	0.71
Tyrosine	109.6	153.1	137.4	137.9	143.2	12.26	0.17	0.21	0.21

^1^Means in the same row with different superscripts significantly differ from one another (*P* <  0.05). *AA* amino acid, *SEM* standard error of the mean; *n* = 6/diet

### Blood chemistry and hematology

No differences in blood chemistry were observed between broilers fed the control and 1% *L*-lysine·H_2_SO_4_ diets (*P* > 0.05) (Table 5). Both albumin and total protein of broilers fed the 7% and 10% *L*-lysine·H_2_SO_4_ diets were higher than those of the broilers fed the control diet (*P* < 0.05), meanwhile increased dietary *L*-lysine·H_2_SO_4_ linearly and quadratically increased albumin and total protein of broiler chickens (*P* < 0.05). Total bilirubin of the broilers fed the 7% *L*-lysine·H_2_SO_4_ diet was higher than that of broilers fed 0, 1%, 4% and 10% *L*-lysine·H_2_SO_4_ diets (*P* < 0.05).

**Table 5:** Effect of dietary *L*-lysine·H_2_SO_4_ levels on serum chemistry of broiler chickens^1^

Items	Dietary *L*-lysine·H_2_SO_4_ levels, %					SEM	*P*-value		
	0	1	4	7	10		ANOVA	Linear	Quadratic
Albumin, g/L	11.1^c^	11.3^c^	12.3^bc^	14.5^a^	13.0^b^	0.38	<0.01	< 0.01	< 0.01
Total protein, g/L	23.7^c^	25.4^bc^	28.2^ab^	29.1^a^	27.7^ab^	0.79	< 0.01	< 0.01	< 0.01
Globulin, g/L	12.6	14.2	15.9	14.6	14.8	0.85	0.13	0.09	0.05
Alanine amino transferase, U/L	21.3^ab^	22.9^ab^	16.8^b^	31.6^a^	23.8^ab^	2.78	0.02	0.15	0.36
Aspartate amino transferase, U/L	65.3	86.9	69.6	68.6	95.1	7.24	0.06	0.18	0.33
Total bilirubin, μmol/L	19.4^b^	22.2^b^	22.7^b^	42.5^a^	20.6^b^	2.90	< 0.01	0.04	0.02
Creatinine, μmol/L	24.0	25.3	26.5	25.2	21.6	1.18	0.12	0.36	0.03
Uric acid, μmol/L	332.2	356.1	291.7	250.5	365.5	46.66	0.43	0.66	0.55
Lactic dehydrogenase, U/L	1508.0	1443.8	1567.5	1146.0	1792.7	182.12	0.24	0.84	0.52

^1^Means in the same row with different superscripts differ from one another (*P* < 0.05). *SEM* standard error of the mean; *n* = 6/diet

No significant differences in hematology were observed between broilers fed the control and 1% *L*-lysine·H_2_SO_4_ diets (Table 6). Hemoglobin and corpuscular hemoglobin concentration of the broilers fed the 10% *L*-lysine·H_2_SO_4_ diet were lower than those of the broilers fed the control and 1% *L*-lysine·H_2_SO_4_ diets (*P* < 0.05). Increasing dietary *L*-lysine·H_2_SO_4_ linearly and quadratically decreased the corpuscular hemoglobin content of broilers (*P* < 0.05).

**Table 6:** Effect of dietary *L*-lysine·H_2_SO_4_ levels on blood hematology of broiler chickens^1^

Items	Dietary *L*-lysine·H_2_SO_4_ levels, %					SEM	*P*-value		
	0	1	4	7	10		ANOVA	Linear	Quadratic
White blood cells, 10^9^/L	49.0^ab^	37.9^b^	48.6^ab^	55.8^a^	54.1^ab^	4.20	0.05	0.07	0.11
Red blood cells, 10^12^/L	2.6^ab^	2.6^ab^	2.7^ab^	2.8^a^	2.5^b^	0.07	0.04	0.91	0.11
Hemoglobin, g/L	86.5^a^	86.6^a^	83.6^a^	89.1^a^	73.0^b^	3.30	0.02	0.06	0.04
Hematocrit, %	33.1^ab^	33.7^ab^	33.0^ab^	34.9^a^	30.1^b^	0.99	0.05	0.26	0.08
Corpuscular volume, fL	127.7^ab^	130.4^a^	121.7^b^	124.7^ab^	122.4^b^	1.81	0.01	0.01	0.05
Corpuscular hemoglobin concentration, g/L	261.1^a^	258.4^a^	252.3^ab^	255.4^ab^	242.2^b^	4.07	0.03	< 0.01	0.01
Red cell distribution width, fL	35.7	37.5	36.3	37.3	38.5	1.40	0.66	0.22	0.47
Platelet distribution width, fL	6.8	6.1	7.1	6.6	6.5	0.59	0.80	0.91	0.99
Platelet volume, fL	8.2	8.8	9.2	9.1	8.3	0.49	0.49	0.65	0.18
Proportion of large platelets, %	15.2	20.5	20.4	18.7	15.4	3.20	0.62	0.98	0.28

^1^Means in the same row with different superscripts significantly differ from one another (*P* < 05); *SEM* standard error of the mean; *n* = 6/diet

## Discussion

*L*-lysine·H_2_SO_4_, as a new source of lysine, contains *L*-lysine·sulphate and fermentation co-products which contain other amino acids and phosphorus [7]. We evaluated effects of *L*-lysine·H_2_SO_4_ on intestinal morphology and liver pathology when used as a source of lysine in broilers diets. We showed that excessive *L*-lysine·H_2_SO_4_ addition in corn-soybean meal diets have negative effects in broilers.

The high proportion of L-lysine·H_2_SO_4_ (4%, 7% and 10%) in diets had different effects on performance and carcass characteristics when compared to the control and 1% *L*-lysine·H_2_SO_4_ supplementation diets. These results are consistent with previous studies that reported excessive lysine supplementation affected feed intake, weight gain, carcass traits, and feed conversion ratio [16–18]. The content and ratio of dietary amino acids affect the efficiency of amino acid utilization for animal growth [19–21]. For example, 1.95% lysine decreased feed intake and growth rate through reducing arginine efficacy in chicks [22]. In young pigs, three to four times the recommended lysine level (1.15% for young pigs) also decreased feed intake and weight gain through aggravating an arginine deficiency [23]. These results indicated that high levels of *L*-lysine·H_2_SO_4_ that reduced growth performance in broilers may down so by lowering the utilization ratio of other amino acids. In addition, excess *L*-lysine·H_2_SO_4_ caused a high level of dietary sulphate which can influence broiler performance via alterations in acid-base balance.

It is generally known that blood plasma proteins and amino acids play key roles in the maintenance of colloid osmotic pressure, by keeping the balance of amino acid through a rapid substitute for indispensable amino acids, assuring steady glucose levels through gluconeogenesis, forming various functional enzymes and enhancing the immune system in the organism [24–26]. Therefore, blood plasma proteins and amino acids have an exceptional significance in maintenance of homeostasis. Changes in serum levels of lysine, threonine, phenylalanine, valine, isoleucine, and leucine in the present experiment were possibly mediated through a change in dietary amino acid levels in the experimental diets.

Blood chemistry is a common parameter utilized to estimate avian body condition. Blood albumin serves as the most favorable source of amino acids for synthesis of tissue proteins in the period of quick somatic growth of birds, especially under feed restricted conditions [27, 28]. Experiments on chickens revealed a relatively constant level of albumin in blood over a period from the 2 to 42 days of age [28]. In our study, content of total protein and albumin in serum were higher with supplementation of 4%, 7% or 10% *L*-lysine·H_2_SO_4_ than the control group, which is probably a direct consequence of a high demand for amino acids for somatic growth in birds fed excess *L*-lysine diets.

The chemistry profile and hematological count in blood of animals fed different diets can indicate the chemical and nutritional value of the diets [29, 30]. Addition of lysine in poultry diets improved immunity of birds and resulted in immunological regulatory action [31, 32]. However, the number of white blood cells in blood was higher for broilers fed the diet with 7% *L*-lysine·H_2_SO_4_ compared with broiler chicks fed the 1% *L*-lysine·H_2_SO_4_ diets, which could lead to the resulting in the secretion of much more cytokine and led to inflammatory reactions [33, 34]. Therefore, the growth performance of broilers supplemented with 4%, 7% or 10% *L*-lysine·H_2_SO_4_ was negatively affected compared with broiler chicks fed control diets during all phases of the experiment. In addition, the corpuscular hemoglobin concentration was also markedly decreased when the diet was supplemented with 4%, 7% or 10% *L*-lysine·H_2_SO_4_. This response suggests that there were toxic effects on the hematological system particularly for red blood cells [35, 36].

The folds in the intestine called villi are an important exchange area for digestion and absorption [37]. Villous height and crypt depth are critical factors affecting the animal’s ability to absorb nutrients [38]. In the current study, there was a significant reduction of villous height and a decline of crypt depth in the duodenum, jejunum and ileum in broilers supplemented with 7% or 10% *L*-lysine·H_2_SO_4_. Similar findings were reported for weanling pigs fed a lysine-deficient diet [39]. The reduction in villus height would reduce the surface area available for absorption of nutrients and this may partially explain the reduced growth performance and reduced feed efficiency in pigs fed the excessive *L*-lysine·H_2_SO_4_ diet.

The liver is the main size for protein synthesis, and liver histology is important in understanding pathological alterations in response to nutritional sources. Studies have shown that the addition of excessive protein in feed can reduce physiological function of the liver, so the lipoprotein in the liver cells cannot be transported in time, resulting in the accumulation of lipoprotein in the liver and liver lesions [40, 41]. In the present study, we found that high levels of *L*-lysine·H_2_SO_4_ might induce hepatic steatosis in broilers. The total protein content of the serum can reflect the ability of the liver to synthesize protein. The present study showed serum total protein content increased with the addition of *L*-lysine·H_2_SO_4_ in poultry diets, and more than 4% *L*-lysine·H_2_SO_4_ groups were significantly higher than the control group. Hence, excessive *L*-lysine·H_2_SO_4_ could cause a certain degree of damage to the liver cells, which is consistent with the results of liver histology in this study.

## Conclusions

Our results show that supplementation with 1% *L*-lysine·H_2_SO_4_ had no negative effects on broilers, while supplementation with 4%, 7% or 10% *L*-lysine·H_2_SO_4_ negatively influenced growth performance, intestinal morphology and liver pathology. Our data indicates that doses higher than 1% dietary *L*-lysine·H_2_SO_4_ resulted in a negative response for broilers.

## Abbreviations

AA: Amino acids
ADFI: Average daily feed intake
ADG: Average day gain
AOAC: Association of Official Analytical Chemists
FCR: The ratio of average daily feed intake to average daily gain
GLM: General liner model
HCl: Hydrogen chloride
HPLC: High performance liquid chromatography
ME: Metabolic energy
SAS: Statistical Analysis System
